# Quantifying intersecting structural racism in the youth criminal justice system: a whole-population linked administrative data study from Manitoba.

**DOI:** 10.23889/ijpds.v7i3.1832

**Published:** 2022-08-25

**Authors:** Marni Brownell, Stephanie Sinclair, Nathan Nickel, Lorna Turnbull, Rick Linden, Rosemary Ricciardelli, Wendy Au, Jennifer Enns, Wendy Au, Mariette Chartier, Marcelo Urquia, Alyson Mahar, Elizabeth Wall-Wieler, Dan Chateau, Matthew Sanscartier, Hygiea Casiano, Farzana Quddus, Drew Lambert, Ivy Ferland

**Affiliations:** 1 University of Manitoba; 2 First Nations Health and Social Secretariat of Manitoba; 3 Memorial University of Newfoundland; 4 Australian National University

## Objectives

Over three decades ago, the Aboriginal Justice Inquiry identified structural racism in Canada’s justice system. Although rates of youth criminal charges and incarcerations have declined substantially since then, it is unclear whether First Nations youth are treated differently than non-First Nations youth for similar offences. Our study addressed this question.

## Approach

This retrospective cohort study of youth born 1991-2001 and living in Manitoba between ages 12-17 used whole-population linked administrative data to identify youth charged with a crime (N=13,543). First Nations youth (n=7,081) were compared with all other Manitoba (AOM) youth (n=6,462) on whether their criminal charge proceeded or was dropped, deferred or diverted. The study applied an intersectionality theoretical framework. Individual (age, type and severity of charge, ever in child protection care, youth and/or mother diagnosed mental disorder, maternal incarceration, number of siblings, urban/rural residence) and social strata (First Nations identity, income, sex) factors were adjusted for using multi-level models.

## Results

After accounting for other factors, First Nations youth had a higher risk of a charge proceeding than AOM (adjusted Relative Risk (aRR) 1.16, 95% CI 1.12-1.20). There was no difference in charges proceeding for male First Nations youth compared with male AOM, whereas among females, the risk was greater for First Nations (aRR 1.31, 95% CI 1.26-1.36). Low income and a history of being in the care of the child protection system increased the risk of charges proceeding for AOM only (aRR 1.16, 95% CI 1.13-1.18; aRR 1.13, 95% CI 1.07-1.20); for First Nations, there was no increased risk of charges proceeding associated with these intersecting factors.

## Conclusion

These findings provide quantitative evidence of the intersecting structural racism in the youth criminal justice system previously identified by First Nations leaders. Future research will follow this cohort to determine whether more judicial sanctions are applied to First Nations youth throughout the justice process, including within criminal courts and corrections.

